# Central Nervous System Parasitosis and Neuroinflammation Ameliorated by Systemic IL-10 Administration in *Trypanosoma brucei*-Infected Mice

**DOI:** 10.1371/journal.pntd.0004201

**Published:** 2015-10-27

**Authors:** Jean Rodgers, Barbara Bradley, Peter G. E. Kennedy, Jeremy M. Sternberg

**Affiliations:** 1 Institute of Biodiversity, Animal Health and Comparative Medicine, College of Medical, Veterinary and Life Sciences, University of Glasgow, Glasgow, Scotland, United Kingdom; 2 Department of Neurology, Institute of Neurological Sciences, University of Glasgow, Glasgow, Scotland, United Kingdom; 3 Institute of Biological and Environmental Sciences, University of Aberdeen, Aberdeen, Scotland, United Kingdom; New York University School of Medicine, UNITED STATES

## Abstract

Invasion of the central nervous system (CNS) by African trypanosomes represents a critical step in the development of human African trypanosomiasis. In both clinical cases and experimental mouse infections it has been demonstrated that predisposition to CNS invasion is associated with a type 1 systemic inflammatory response. Using the *Trypanosoma brucei brucei* GVR35 experimental infection model, we demonstrate that systemic delivery of the counter-inflammatory cytokine IL-10 lowers plasma IFN-γ and TNF-α concentrations, CNS parasitosis and ameliorates neuro-inflammatory pathology and clinical symptoms of disease. The results provide evidence that CNS invasion may be susceptible to immunological attenuation.

## Author Summary

African trypanosomiasis (also known as sleeping sickness) is caused by the protozoan parasites *Trypanosoma brucei rhodesiense* and *T*.*b*.*gambiense*. After infection through the bite of the tsetse fly vector, parasites initially disseminate in the blood and lymphatic systems, in what is known as the early or haemolymphatic stage of infection. However, as infection progresses some parasites enter the brain, initiating the late or meningoencephalitic stage of infection. This is a critical step in the progression of disease. In rodent model infections, inflammatory cytokine and chemokine responses associated with infection have been demonstrated to promote invasion of the brain and we predicted that this process may be inhibited by administration of the counter-inflammatory cytokine IL-10 during the early stage of infection. We demonstrate that in IL-10-treated mice, trypanosome invasion of the brain is diminished and neuro-inflammatory pathology is reduced. As evidence suggests that systemic inflammatory responses are also associated with promotion of CNS invasion in human patients, we believe that our results offer the possibility of new treatments to delay brain involvement and to identify individuals at high risk of neuroinflammatory complications.

## Introduction

Human African trypanosomiasis (HAT) is caused by two subspecies of *Trypanosoma brucei*; *T*.*b*. *gambiense* and *T*.*b*.*rhodesiense* and presents with typically chronic and acute infection profiles in West and East Africa respectively. The disease progresses through two clinical stages. In the early (stage 1; haemolymphatic) stage parasites are primarily located in the blood and lymphatic vasculature; in the late (stage 2, meningoencephalitic) stage parasites penetrate the blood brain barrier and proliferate in the central nervous system (CNS). There is considerable variation in the tempo of disease progression between and within each of the two subspecies of *Trypanosoma brucei* that cause HAT [1].

Invasion of the CNS is a critical stage in disease progression, and an understanding of the factors that control this process will offer possibilities for therapeutic intervention. The onset of the CNS stage of HAT has been modelled using experimental mice. It has been demonstrated that CNS invasion is promoted by a type 1 inflammatory immune response and the interaction of both TNF-α [2]and IFN-γ and CXCL10 [3]with the brain vascular endothelium. This clinical relevance of this finding is supported by observations in *T*.*b*.*rhodesiense* HAT patients, where high systemic IFN-γ levels were associated with late stage diagnosis[4], and high systemic counter-inflammatory cytokine levels were associated with a delayed onset of the late stage[5]. The involvement of the host-response in driving disease progression is consistent also with studies of peripheral pathogenesis in mouse models where a switch from an early IFN-γ driven Type 1 immune response (essential for initial parasitaemia control) to an IL-10 dominated Type 2 response is essential for extended survival and amelioration of systemic pathologies such as anaemia, cachexia, and liver necrosis[6]. The importance of IL-10 in controlling systemic inflammatory pathology in the mouse model is evident in the severe outcome of infections in IL-10 gene deleted mice [6] and conversely in extended survival and the amelioration of inflammatory pathology in chronically infected mice expressing high levels of virally delivered IL-10 [7]. As systemic (i.v.) delivery of IL-10 has been shown to inhibit inflammatory responses in clinical trials [8], we aimed to determine if systemic IL-10 treatment in an experimental animal model of HAT could delay the invasion of the CNS by trypanosomes and result in reduced CNS pathology.

In this study *T*.*b*.*brucei*-infected mice were systemically treated with recombinant murine IL10[9,10] to test the hypothesis that treatment would reduce pathology and CNS parasite load in a highly defined model of late stage infection[11].

## Materials and Methods

### Animals, infections and treatments

**Fig 1:**
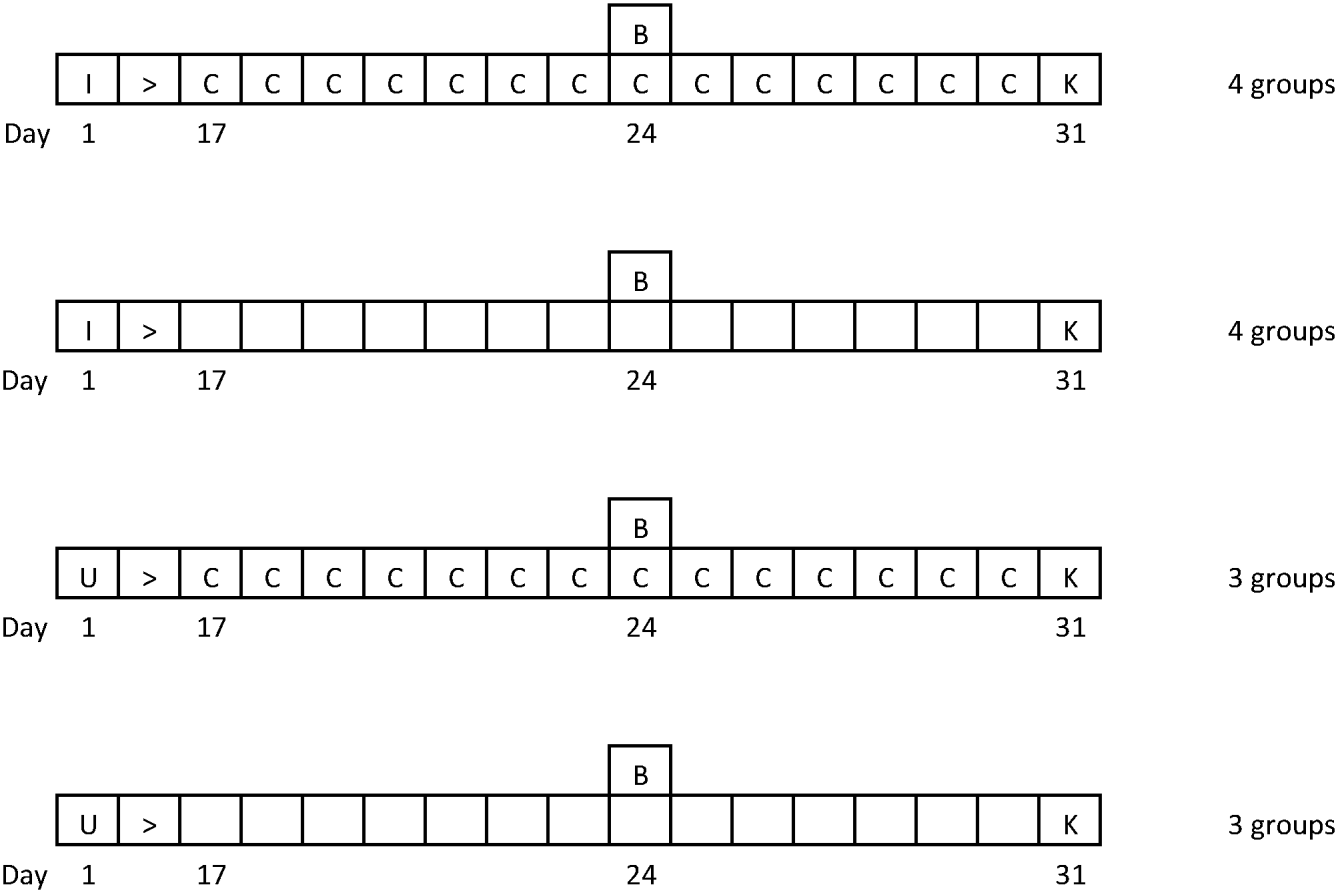
Experimental infection design. Mice were infected with *T*.*b*.*brucei* GVR35 at day 1 (I) and treated with diminazene aceturate (to eliminate the peripheral infection) at day 24 (B). IL-10 treatment was carried out from day 17–30 (B) and all mice were euthanized at day 31 (K) for analysis of neuropathology and parasitosis. Uninfected animals following identical regimens to the infected mice were included as controls (U).

All animal procedures were authorised under the Animals (Scientific Procedures) Act 1986, Home Office Licence PPL60/4414 and approved by the University of Glasgow Ethical Review Committee. Animal treatments and group sizes are presented in detail in Fig 1. Briefly, 8 groups of 6 female CD1 mice (Charles River Laboratories, UK) were infected with 2 x 10^4^ *Trypanosoma brucei brucei* GVR 35 parasites by intraperitoneal injection (i.p.). 4 of the groups were given 4μg of recombinant murine IL-10 (PeproTech EC London) i.p. daily for 14 days, beginning on day 17 post-infection. A further 6 groups of uninfected control animals were maintained, of which 3 groups were treated with the IL-10 administration regimen described above. On day 24 post-infection all (infected and control) mice were treated with 40mg/kg diminazene aceturate i.p. (Berenil, Hoechst, Germany) to exacerbate the CNS inflammatory reaction. Infection was confirmed by examination of a wet blood film prior to treatment. On day 31 post-infection the mice were sacrificed and peripheral blood removed from the brain by transcardial perfusion with 120 mL of sterile saline and the brain excised for further analysis.

### Clinical assessment and neuropathological analysis

Each mouse was examined daily to evaluate the degree of clinical impairment using an established visual assessment scale [12] that progresses incrementally from 0–6 as the severity of the clinical response increases.

At necropsy the excised brains were fixed in neutral buffered formalin and embedded in paraffin wax prior to sectioning. The severity of the neuro-inflammatory reaction was assessed in haematoxylin and eosin stained sections using a predefined scoring system where 0 indicates no pathological changes and 4 denotes the presence of a severe meningoencephalitis [12].

### Quantitative PCR

Trypanosome load within the brain was determined using Taqman real-time PCR as previously described [13]. Briefly, following sacrifice brains were immediately frozen on solid carbon dioxide and stored at -70°C until required. DNA was prepared from a 25 mg sample of whole brain homogenate (Qiagen, DNeasy Tissue kit). Taqman PCR to detect the trypanosome *Pfr*2 gene was performed in a 25 μL reaction mix comprising 1 x Taqman Brilliant II master mix (Agilent, UK), 0.05 pmol/μL forward primer (ccaaccgtgtgtttcctcct), 0.05 pmol/μL reverse primer (gaaaaggtgtcaaactactgccg), 0.1 pmol/μL probe (fam-cttgtcttctccttttttgtctctttccccct-tamra) (Eurofins MWG Operon, Germany) and 100 ng template DNA. The amplification was performed on a MxPro 3005 (Agilent) with a thermal profile of 95°C for 10 minutes followed by 45 cycles of 95°C for 15 seconds, 60°C for 1 minute and 72°C for 1 second.

A standard curve was constructed using a serial dilution (range; 1 x 10^6^ to 1 x 10^1^ copies) of pCR2.1 vector containing the cloned *Pfr*2 target sequence (Eurofins MWG Operon) and the trypanosome load within the brain samples was calculated using the MxPro qPCR software (Agilent).

### Cytokine assays

Plasma cytokine concentrations were measured using solid phase sandwich ELISA (BD OptEIA, B-D Bioscience, Oxford UK) as described in [14]. The detection limits for each assay were IFN-γ: 10pg/ml, IL-10: 50pg/ml, IL-6: 5pg/ml, TNF-α: 16pg/ml.

### Statistical analysis

Univariate analysis of necropsy data was carried out using 1 way ANOVA with Tukey’s post-hoc test or Student’s t-test as appropriate. Development of clinical impairment was assessed using 2-way Repeated Measures ANOVA.

## Results

### IL-10 treatment reduces the development of neuroinflammation and CNS parasitosis in late stage African trypanosome infection

**Fig 2:**
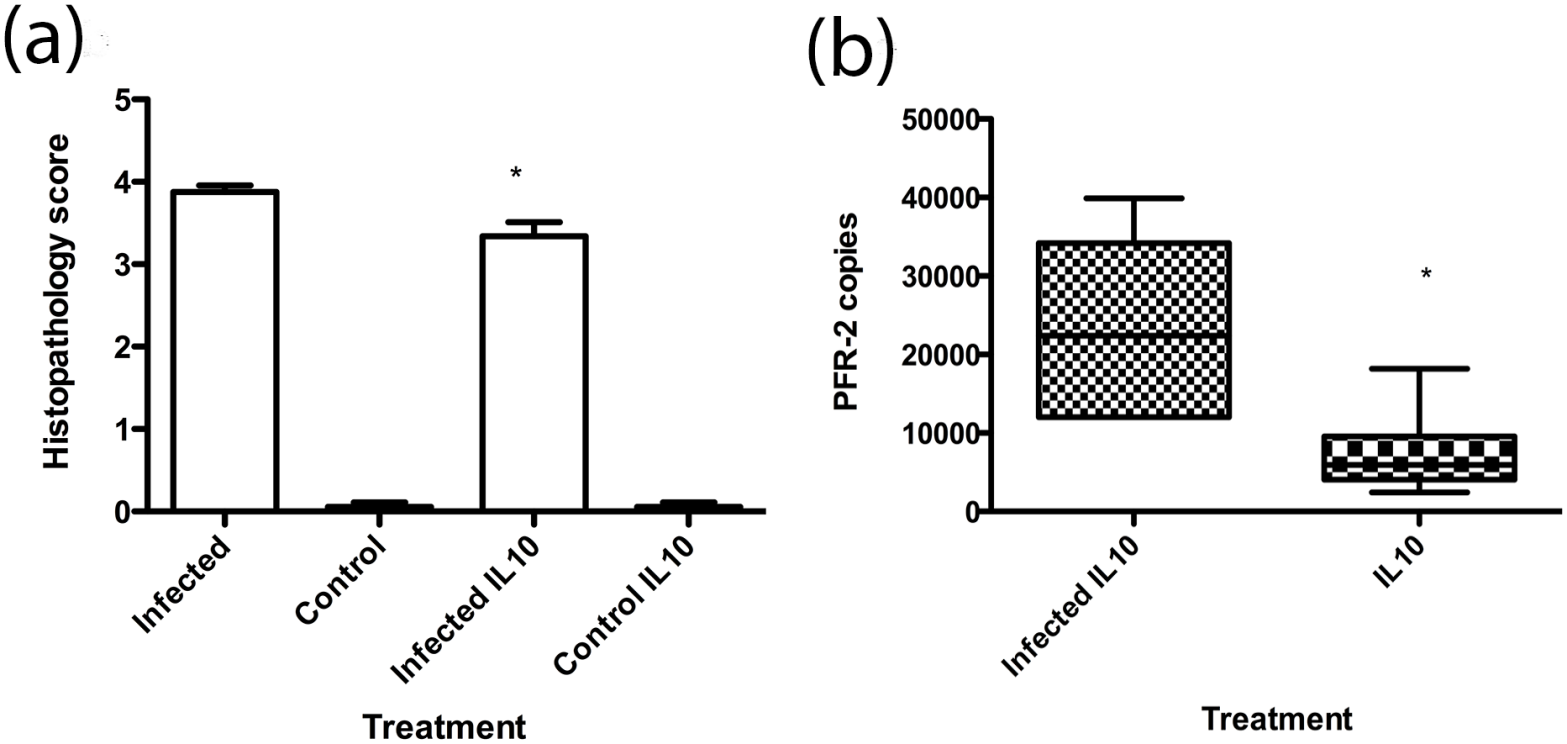
IL-10 administration reduces neuropathology and CNS parasitosis in experimental African trypanosome infection. (a) Neuropathology scores were measured in infected (n = 5), control (n = 6), infected and IL-10 treated (n = 5) and IL-10 treated controls (n = 6). Data are mean ±SE. *:p<0.05 Tukey post-hoc test (b) CNS parasitosis in IL-10 treated (n = 6) and untreated infected mice (n = 6) was measured by Q-PCR. Boxes are median and IQR, whiskers 10^th^ and 90^th^ percentile. *:P<0.05 Unpaired *t-*test with Welch correction.

In the late stage infection model, mice are given a sub-curative treatment with diminazene aceturate 24 days after-infection with *Trypanosoma brucei*. This clears (temporarily) the trypanosomes from the peripheral compartment but allows continued trypanosome proliferation in the brain to the experimental end point at day 31 post-infection and instigates a severe neuroinflammatory reaction[15]. In the IL-10 treatment groups, mice were given 4μg recombinant murine IL-10 daily from day 17 post-infection. Histological examination of the brains of uninfected control mice with or without IL-10 administration indicated no deviation from normal histology (Fig 2a). While, as expected, both IL-10 treated and untreated infected mice exhibited significant increases in neuropathology, those treated with systemic IL-10 from day 17 of infection exhibited a significantly lower neuropathological grade (3.34±0.08) compared to untreated infected mice (3.87 ± 0.17, mean ± SE, *p*<0.05 Tukey post-hoc test). Taqman PCR analysis revealed that IL-10 treatment significantly (p<0.01) reduced the *Pfr*2 copy number (7302 ± 2261) detected within the brain of infected animals compared with non-IL10 treated mice (23507 ± 4863) (Fig 2b).

### IL-10 treatment reduces systemic IFN-γ and TNF-α levels

**Fig 3:**
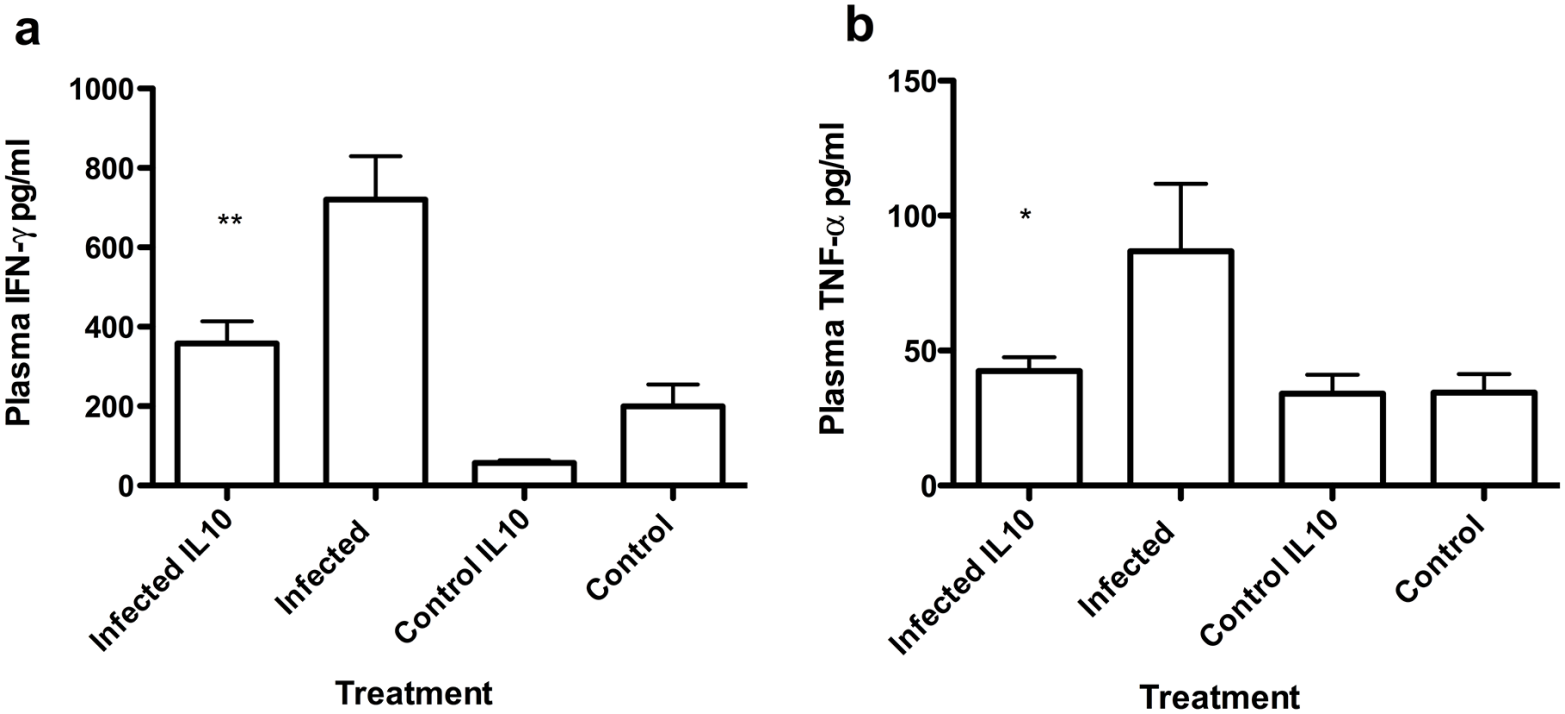
Effect IL-10 administration on plasma cytokine concentrations. (a) Plasma IFN-γ and (b) Plasma TNF-α concentrations in infected (n = 17), control (n = 8), infected and IL-10 treated mice (n = 17) and IL-10 treated controls (n = 8). Data are mean+SE *p<0.05; **:p<0.01 tukey post-hoc test.

At the experimental end point plasma samples were taken to determine circulating cytokine levels. While no significant differences were observed in IL-6 and IL-10 concentrations (S1 Table), the elevation of plasma IFN-γ concentration observed in both infection groups was significantly reduced in IL-10-treated infected mice (Fig 3a) and plasma TNF-α concentrations were reduced to control levels (Fig 3b).

### IL-10 treatment reduces the onset of clinical symptoms

**Fig 4:**
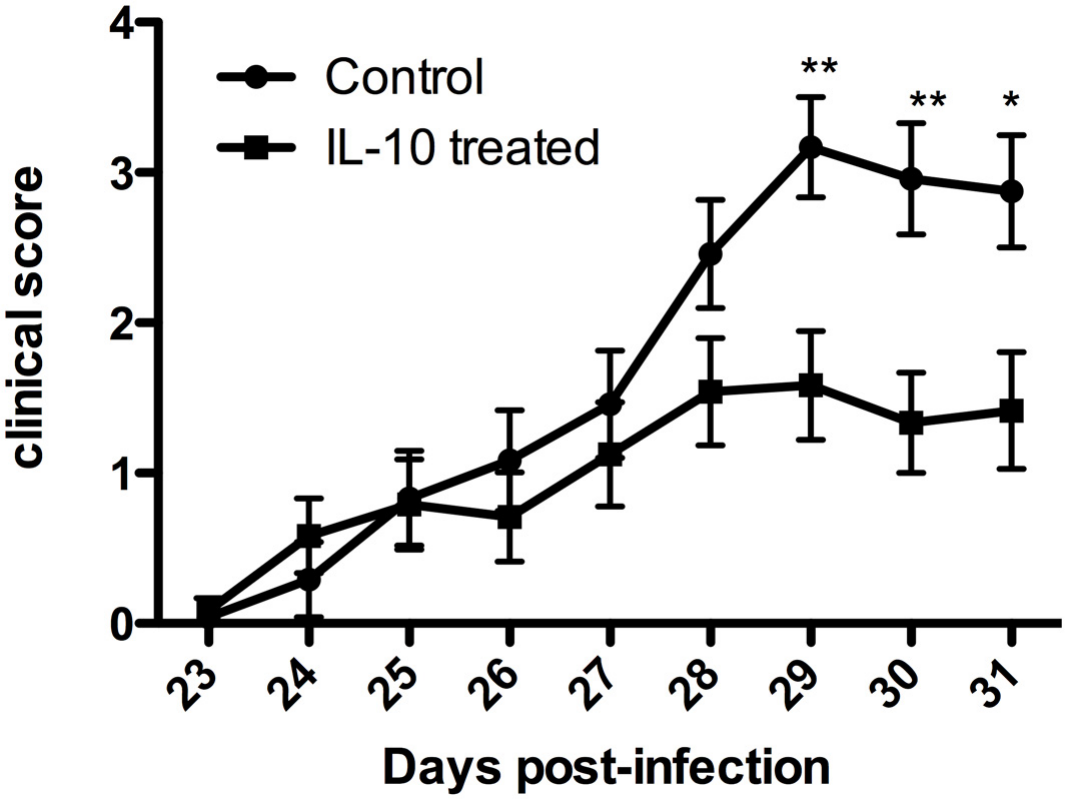
IL-10 treatment reduces clinical symptoms in late stage model. IL-10 treated (n = 24) and control (n = 24) were assessed daily for clinical score. Data are mean±SE. No clinical signs were observed before day 24 post-infection. Significant reduction in clinical score **: p<0.01 *: p<0.05 Bonferonni corrected post hoc test.

Clinical scores in individual mice were monitored daily (Fig 4). No clinical signs were associated with the early stages of the experiment. Between days 23 and 27 post infection clinical signs were noted in both IL-10 treated and non-treated infected animals. However the mean response in both groups was similar and remained low (<1.5). There was a slight increase in clinical score at day 28 of infection in the non-treated group, and this then became a marked and significant increase (Repeated Measures ANOVA Bonferroni post-hoc test) at days 29 (p<0.01), 30 (p<0.01) and 31 (p<0.05). Thus, the infected mice that did not receive IL-10 treatment exhibited a significantly more severe clinical response compared with the IL-10 treated animals after day 29 of infection, while the maximum mean clinical score in the IL-10 treated mice was less than 1.6.

## Discussion

Invasion of the CNS is a critical step in the progression and morbidity of human African trypanosomiasis. Evidence from experimental models suggests that the process depends on a systemic inflammatory response, particularly involving IFN-γ and TNF-α; this is consistent with the relationship of plasma IFN-γ levels and the development of meningoencephalitic infection observed in clinical subjects and leads to the prediction that down-regulation of the systemic inflammatory response would limit the invasion of the CNS and therefore ameliorate CNS pathology.

Using an established and highly reproducible model of late stage HAT in experimental mice, we demonstrate that administration of the counter-inflammatory cytokine IL-10 leads to a decrease in CNS parasite burden and neuropathology. Furthermore, this treatment significantly improved the clinical response of the mice to trypanosome infection. Symptomatic amelioration occurs from day 28 of infection, and this is coincident with the time of moderate to severe neuro-inflammatory response in this experimental model[14]. At the experimental end point (day 31 post-infection) there was a reduction in perivascular cuffing and encephalitis contributing to a significantly improved neuropathology score in IL-10 treated mice. This probably was a consequence of the lower CNS parasite burden in IL-10 treated mice. IL-10 treated mice also exhibited reductions in systemic plasma IFN-γ and TNF-α concentrations. The degree of reduction of TNF-α is consistent with previous experimental studies in which exogenous IL-10 has been used to modulate endotoxic shock[9], and the reduction in IFN-α concentration is consistent with the known antagonistic role of IL-10 to Th1 cytokine responses in experimental trypanosomiasis[6]. As the terminal plasma samples were taken 24h after IL-10 administration, the lack of any additional IL-10 being detected is most likely due to the rapid turnover of IL-10. This has been demonstrated in humans[16] and experimental mice where plasma levels have been shown to fall from 250pg/ml to below 30pg/ml in a little as 3 hours [17]. Because IFN-γ and TNF-α promote cellular interactions that facilitate the invasion of the CNS from the vasculature by trypanosomes and thus regulate subsequent parasitosis [2,3], we propose that the down-regulation of these cytokines after IL-10 administration is the primary mechanism underlying the effect of IL-10 treatment observed here. Importantly a strong association has also been shown in clinical studies of HAT between the probability of rapid progression to CNS infection and increased plasma IFN-γ concentrations[18], suggesting that these results may be extrapolated to the development of the meningoencephalitic stage of the clinical disease.

The application of IL-10 treatment from day 17 of infection in this study is based on pathological and pharmacological evidence that *T*.*brucei* GVR35 establishes within the brain parenchyma between 14 and 21 days post-infection [19]. There have been recent reports of trypanosomes in the CNS at very early times after infection in similar experimental models using intra-vital imaging [20], but the significance of such observations in relation to our study remain unclear given that infections at an early stage are effectively cleared using drugs (such as diminazene aceturate) that do not cross the blood brain barrier [19]. While experimental administration of non-steroidal anti-inflammatory agents [11] and Substance-P antagonists [21] have previously been shown to reduce neuropathology in this late stage model validating the principle of inflammatory modulation of meningo-encephalitis, no data on the modulation of CNS parasitosis were available. Similarly, the concept of IL-10 modulation of systemic pathology was confirmed by the use of adenoviral vector delivery of IL-10 [7]. Our study demonstrates the potential for modulation of the most critical step in the progression and pathogenesis of clinical African trypanosome infections, namely the invasion of the CNS and development of neuropathology, using IL-10. It seems unlikely that this finding could be directly applied to the treatment of HAT despite the fact that IL-10 therapy is well tolerated and may be effective in other chronic pathologies involving inflammatory dysregulation[22]. Rather we see these findings as demonstrating the potential for effective treatments using more refined immuno-therapeutics and further emphasising the importance of host-inflammatory response polymorphisms in understanding the diverse spectrum of progression observed in HAT.

## Supporting Information

S1 Table Plasma IL-10 and IL-6 concentrations at experimental end point. (DOCX)
