# Supplementary material for: Central Nervous System Parasitosis and Neuroinflammation Ameliorated by Systemic IL-10 Administration in Trypanosoma brucei-Infected Mice
Source: PLoS Negl Trop Dis. 2015 Oct 27;9(10):e0004201. doi: 10.1371/journal.pntd.0004201 (PMC4624684; doi:10.1371/journal.pntd.0004201)
Supplement: S1 Table — (DOCX) [file pntd.0004201.s001.docx]

S1 Table.

Plasma IL-10 and IL-6 concentrations at experimental end point

| Treatment | Plasma IL-6 pg/ml (mean±se) | Plasma IL-10 pg/ml (mean±se) |
| --- | --- | --- |
| Infected n=17 | 34.3±6.4 | 115.7±17.1 |
| Infected-IL10 treated n=17 | 20.7±3.2 | 131.7±26.9 |
| Control n=8 | 31.3±13.7 | 91.3±15.7 |
| Control IL-10 treated n=8 | 11.1±1.0 | 81.5±29.8 |
